# Why Does It Shine?—A Prognostic Analysis about Predisposing Factors for Blood–Brain Barrier Damage after Revascularisation of Cerebral Large-Vessel Occlusion

**DOI:** 10.3390/jcdd10050185

**Published:** 2023-04-22

**Authors:** Michael Knott, Stefan Hock, Liam Soder, Iris Mühlen, Svenja Kremer, Maximilian I. Sprügel, Jochen A. Sembill, Joji B. Kuramatsu, Stefan Schwab, Tobias Engelhorn, Arnd Doerfler

**Affiliations:** 1 Department of Neuroradiology, Friedrich-Alexander-Universität Erlangen-Nürnberg (FAU), Schwabachanlage 6, 91054 Erlangen, Germany; 2 Department of Neurology, Friedrich-Alexander-Universität Erlangen-Nürnberg (FAU), Schwabachanlage 6, 91054 Erlangen, Germany

**Keywords:** flat-detector CT (FDCT), acute ischaemic stroke (AIS), large-vessel occlusion (LVO), endovascular treatment (EVT)

## Abstract

Background: Hyperdense lesions in CT after EVT of LVO are common. These lesions are predictors for haemorrhages and an equivalent of the final infarct. The aim of this study based on FDCT was the evaluation of predisposing factors for these lesions. Methods: Using a local database, 474 patients with mTICI ≥ 2B after EVT were recruited retrospectively. A postinterventional FDCT after recanalisation was analysed regarding such hyperdense lesions. This was correlated with a variety of items (demographics, past medical history, stroke assessment/treatment and short-/long-term follow-up). Results: Significant differences were present in NHISS at admission, regarding time window, ASPECTS in initial NECT, location of the LVO, CT-perfusion (penumbra, mismatch ratio), haemostatic parameters (INR, aPTT), duration of EVT, number of EVT attempts, TICI, affected brain region, volume of demarcation and FDCT-ASPECTS. The ICH-rate, the volume of demarcation in follow-up NECT and the mRS at 90 days differed in association with these hyperdensities. INR, the location of demarcation, the volume of demarcation and the FDCT-ASPECTS could be demonstrated as independent factors for the development of such lesions. Conclusion: Our results support the prognostic value of hyperdense lesions after EVT. We identified the volume of the lesion, the affection of grey matter and the plasmatic coagulation system as independent factors for the development of such lesions.

## 1. Introduction

Endovascular treatment (EVT) has an important role in today’s therapy for patients who have suffered acute ischemic strokes (AISs) due to large vessel occlusion (LVO). Based on multiple randomised trials and everyday clinical experience, it has become standard of care in the management of patients who have had AISs and LVO [1,2,3,4,5,6,7,8,9,10].

After successful thrombectomy, a whole-brain flat-detector CT (FDCT) or conventional CT is established in the clinical workflows of many institutions that perform EVT. These examinations are normally performed without secondary administration of contrast media and are performed to screen for the haemorrhagic complications associated with the previous treatments as well as for an early assessment of stroke extension [11,12].

FDCT, as a method of on-site CT-like imaging in the angiographic suite, is becoming the dominant imaging modality for these examinations not only from the practical point of reduction in secondary patient transfers but also due to improvements in FDCT quality. FDCT is becoming almost equivalent to non-enhanced CT (NECT) in diagnostic criteria regarding the detection of infarct signs and haemorrhages [11,12,13,14,15].

The hyperdense lesions in postinterventional CT imaging have been described since the first cases of endovascular treatments of LVO. The pathophysiology behind this phenomenon is ischemic damage to blood–brain barrier, which leads to the extravasation of contrast agent administered during the EVT. These hyperdense lesions are seen as predictors for a later haemorrhagic transformation of ischemic brain tissue. Furthermore, these hyperdense areas demonstrate a correlation with the minimal size of the final infarct extension [16,17,18,19,20,21,22,23].

Both the higher risk of a haemorrhagic transformation as well as the minimal size of the final infarct may have an impact on clinical decisions in this early phase after the acute treatment of LVO. However, in order to truly utilise this information, radiologists and neurologists alike have to better understand this phenomenon as at the moment the question regarding influencing factors for the actual development of these hyperdensities remains unanswered.

Therefore, the aim of our study based on early FDCT after EVT was to analyse the predisposing factors for the development of hyperdense lesions using a large cohort of patients as well as a variety of clinical items in order to better understand this phenomenon in clinical practice.

## 2. Materials and Methods

### 2.1. Patients

The patient recruitment for this study was based on the STAMINA (Stroke Research Consortium in Northern Bavaria; www.clinicaltrials.gov, accessed on 19 April 2023; NCT04357899) database, a longitudinal cohort study for patients who have had ischemic strokes and been admitted to the University Hospital Erlangen, Germany, beginning in 1 January 2006. The study gained approval from the local institutional review board, Friedrich-Alexander-University Erlangen-Nuremberg, Germany (Registration No 62_21B). Patients or legal representatives provided informed consent.

**Figure 1:**
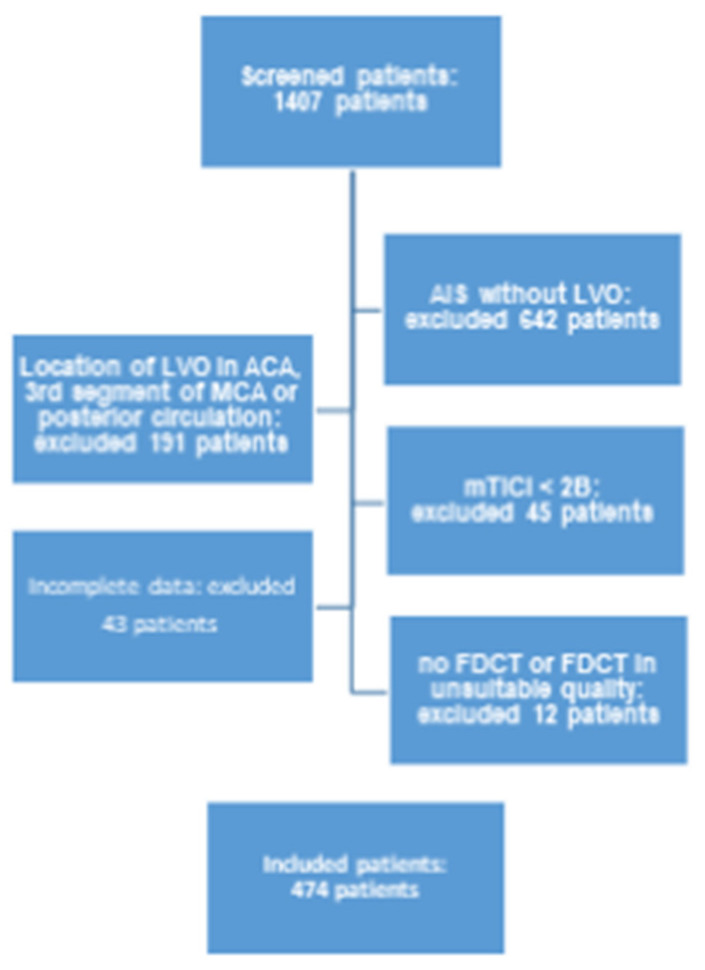
Flowchart of inclusion criteria of this study.

This study includes 474 patients from the abovementioned database, who fulfilled the following inclusion criteria (Figure 1):

1. Period of treatment between 1 January 2015 and 30 June 2019.
2. AIS and intracranial LVO of the anterior circulation (namely the internal carotid artery (ICA) as well as the M1 and M2 segments of the middle cerebral artery (MCA)) with successful EVT (mTICI > 2B).
3. Complete clinical and radiological documentation for each individual patient (a full overview of involved items is given in Table 1).
4. FDCT with adequate diagnostic quality acquired directly after EVT (evaluated by two experienced neuroradiologists (HS and KM)).

No preselection was carried out in regard to the content of the FDCT or regarding clinical data.

### 2.2. Clinical and Radiological Data

The collection of data included demographics, data regarding past medical history (including comorbidities and medication), stroke assessment (NIHSS, time window and data from the initial imaging) and stroke treatment (IV thrombolysis, EVT-related data) as well as short- and long-term follow-up data (follow-up CT at 24–48 h, mRS at 90 days). The detailed summary of clinical test items is given in Table 1. All data were extracted from the STAMINA database, which contains information from clinical reports during the admission of the patients as well as routinely acquired follow-up data.

Both the diagnostic CT imaging prior to EVT as well as the follow-up imaging were acquired via a commercially available scanner (Siemens Somatom Definition AS+; Siemens Healthineers, Forchheim, Germany) using commercially available software (syngo.via, Siemens Healthineers, Erlangen, Germany). Dedicated automatic analysing software was used for a systematic and standardised approach to ASPECTS (RAPID, iSchema View inc, Menlo Park, CA, USA) and CT perfusion evaluation (Brainomix, e-CTP (Brainomix Ltd., Oxford, UK).

### 2.3. FDCT

For all 474 patients included in this study, FDCT examination was performed in the angiographic suite directly after EVT. This imaging covered the whole brain and did not involve additional administration of a contrast medium for the FDCT. All FDCT data were acquired via dedicated angiographic systems (Axiom Artis dBA or Axiom Artis Zee Zeego; Siemens Healthineers, Forchheim, Germany) using the 20sDR-H programme (DynaCT, Siemens AG Healthcare Sector, Forchheim, Germany; acquisition time 20 s, matrix 512, projection on 30 × 40 cm flat-panel size, total angle 217°). Reconstruction was performed in transversal angulation (angulated in relation to the base of the skull) with a slice thickness of 4.8 mm and without gaps between slices. Post-processing took place in an optimised soft tissue kernel (W380 C80).

### 2.4. Imaging Analysis

Syngo.via software (syngo.via, Siemens Healthineers, Erlangen, Germany) was used for radiological analysis. This analysis was carried out by two experienced neuroradiologists (HS and KM) with 7 and 9 years of neuroradiological and neurointerventional experience. Imaging analysis involved:

1. Presence of hyperdense areas in FDCT.
2. Analysis of hyperdense lesions in FDCT regarding:
  - volume of the hyperdensity;
  - involved brain regions (basal ganglia vs. white matter vs. cortical);
  - categorising by appliance of ASPECTS.

As commercial software for automatic volumetric analysis and analysis regarding ASPECTS in FDCT is not yet available, these assessments were performed manually and with the consent of the abovementioned neuroradiologists. Volumetric analysis was carried out by defining the affected area slice by slice and adjusting this result with the thickness of the slice.

### 2.5. Data Analysis

For statistical analysis, commercially available software (BM^®^ SPSS^®^ Statistics Version 28, Chicago, IL, USA) was used. Testing for normal distribution of all data was carried out via the Shapiro–Wilk test. In all cases, non-normal distribution was present. Parameters are presented as median and interquartile ranges. Testing of all clinical and radiological parameters regarding a correlation with the presence of hyperdensities in FDCT after EVT was performed via chi-square test for dichotomised variables and otherwise via the Mann–Whitney U test.

Statistical significance was assumed for a *p* value of less than 0.05.

Spearman’s rank correlation coefficient was used for further analysis of these correlations.

Items with statistically significant values were than tested using a multivariable regression analysis for independent influence on the dependent variable.

For parameters with a *p* value of <0.05, a receiver operating characteristic (ROC) curve analysis with a calculation of the area under the curve (AUC) was carried out to determine the predictive power.

## 3. Results

### 3.1. Basic Charcteristics

In total, 474 patients were included in this study (266 female (56.1%) and 208 male (43.9%); 75.2 [61.9–82.2] years). LVO was on the left side in 232 cases (48.9%). The most proximal occluded vessel was the ICA in 140 cases (29.5%), the M1 segment of the MCA in 249 cases (52.5%) and the M2 segment in 85 cases (17.9%). The initial NHISS was 15 [11–19]. mRS at admission was 0 [0–2], which changed after 90 days to 4 [2–5].

### 3.2. Hyperdensity in Patient Context

**Figure 2:**
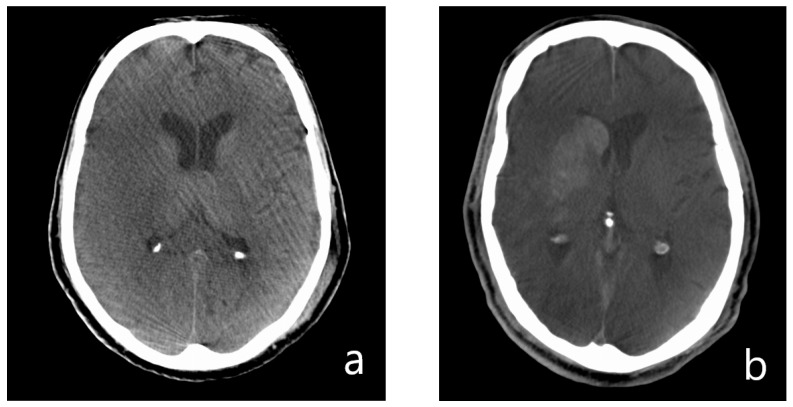
Representative examples of FDCT after EVT. (**a**) Hypodense pattern of demarcation in the MCA territory on the right. (**b**) Hyperdense form of demarcation in the basal ganglia on the right side with contrast extravasation after EVT.

After EVT, 288 patients (60.8%) had hyperdense lesions in FDCT, whereas 186 patients did not have such lesions (28 patients (5.9%) had hypodense lesions, 158 patients (33.3%) did not show any sign of demarcation). Representative FDCT are shown in Figure 2. Full data on all items is shown in Table 1.

### 3.3. Demographics

The two groups showed no significant difference in age (72.4 [63–82.7] vs. 74.9 [61.8–81.5] years; *p* = 0.549) and gender (female: 56.6% vs. 55.4%; male: 43.4% vs. 44.6%; *p* = 0.794).

### 3.4. Past Medical History

Both groups also did not differ in a statistically significant way in the presence of arterial hypertension (79.5% vs. 78%; *p* = 0.712) and diabetes (29.9% vs. 30.1%; *p* = 0.963) as well as in the present medication at admission regarding anti-platelet therapy (mono-therapy: 25% vs. 29%; dual-therapy: 2.4% vs. 2.2%; *p* = 0.409) and anticoagulation (21.5% vs. 16.1%; *p* = 0.145).

### 3.5. Stroke Assessment

The two groups differed in a statistically significant way in the NHISS at admission (16 [12–20] vs. 14 [10–20], *p* < 0.001, Spearman-rho: 0.166) and in the known time window (173 min [68.8–426.3] vs. 96.5 min [60–263.3], *p* = 0.002, Spearman-rho: 0.15). Blood samples showed significant differences between our subgroups in haemostatic parameters (INR: 1.1 [1.0–1.3] vs. 1.1 [1.0–1.2], *p* = 0.019, Spearman-rho: 0.108; INR (rating); tendency towards too high in the hyperdense group (too high values 22.6% vs. 15.6%), *p* = 0.046; Spearman-rho: 0.092; aPTT (rating); and tendency towards too high in the hyperdense group (too high values 8% vs. 2.7%), *p* = 0.016, Spearman-rho: 0.111).

Regarding imaging, both groups did have a statistical difference in the initial ASPECTS in NECT (8 [7–10] vs. 9 [8–10], *p* < 0.001, Spearman-rho: −0.211), the location of the most proximal LVO (tendency towards more proximal LVO in the hyperdense group (ICA occlusion: 36.5% vs. 18.8%), *p* = 0.001, Spearman-rho: −0.146) and CT perfusion ((65.6% vs. 76.3%), *p* = 0.013, Spearman-rho: −0.114; infarct core: 12 mL [0–41] vs. 0 mL [0–12], *p* < 0.001, Spearman-rho: 0.287; penumbra: 133 mL [92–186] vs. 108 mL [56–166], *p* = 0.007, Spearman-rho: 0.15; mismatch ratio: 4 [2.8–9.5] vs. 8.2 [4.3–12.5], *p* = 0.005, Spearman-rho: −0.21).

### 3.6. Stroke Treatment

Both subgroups did not show significant differences in the rate of IV thrombolysis (63.5% vs. 71.5%; *p* = 0.081). Whereas, the following *EVT-related* items demonstrated statistically significant differences: duration of EVT (72 min [48.3–107.8] vs. 55.5 min [9–82.5], *p* < 0.001, Spearman-rho: 0.203), the number of EVT attempts (2 [1–3] vs. 2 [1–3], *p* = 0.004, Spearman-rho: 0.131) and the TICI score (higher percentage of TICI 2B in the hyperdense group (27.1% vs. 17.7%), *p* = 0.019, Spearman-rho: −0.108).

### 3.7. FDCT after EVT

The results of the FDCT after EVT differed in the type of brain region affected (dominance in areas of grey matter in the hyperdense group, *p* < 0.001, Spearman-rho: 0.791), the volume of demarcation (25.1 mL [2.8–33.7] vs. 0 mL [0–0], *p* < 0.001, Spearman: 0.751) and the FDCT-ASPECTS (8 [7–9] vs. 10 [10–10], *p* < 0.001, Spearman-rho: −0.769).

### 3.8. Follow-Up

In the *follow-up NECT* (24–48 h after EVT), both groups showed statistical difference in the rate of ICH (13.9% vs. 4.8%, *p* = 0.002, Spearman-rho: 0.145) and the volume of demarcation (54.5 mL [18–130] vs. 8 mL [0–27.9], *p* < 0.001, Spearman-rho: 0.451). The mRS at 90 days also differed between our subgroups (4 [3–6] vs. 3 [2–4]; *p* < 0.001).

### 3.9. Multivariable Regression Analysis

**Figure 3:**
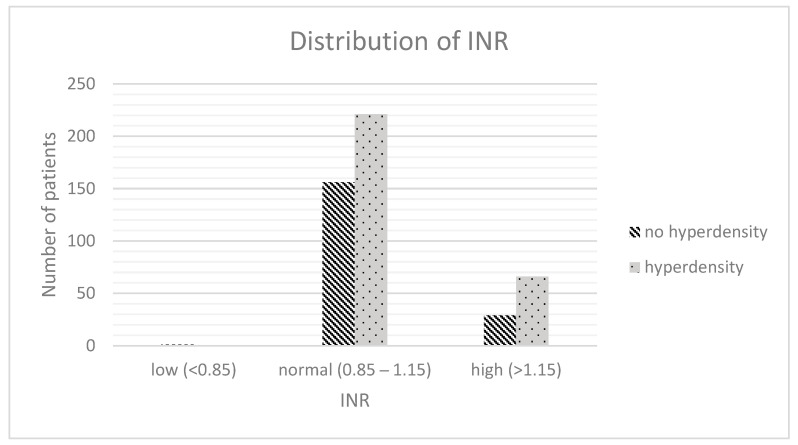
Distribution of INR.

**Figure 4:**
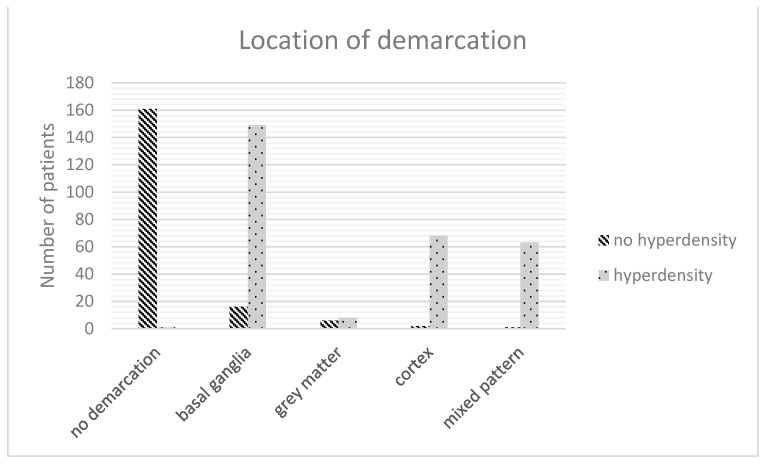
Distribution of infarct location.

**Figure 5:**
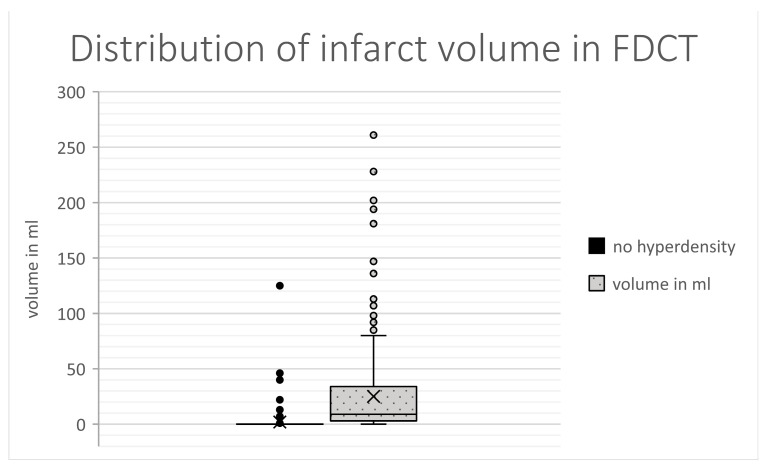
Distribution of infarct volume.

**Figure 6:**
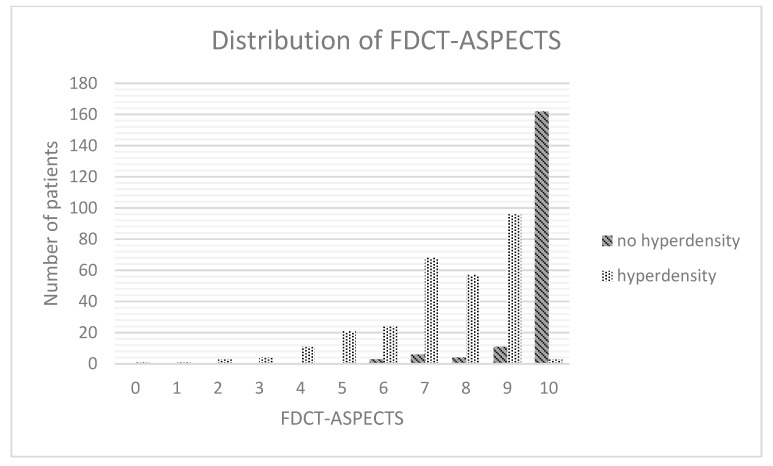
Distribution of FDCT-ASPECTS.

Multivariable regression analysis of the abovementioned statistically differing items revealed the INR (*p* = 0.003, OR: 1.212, 95%-CI: 1.067–1.379; Figure 3), the location of demarcation (*p* < 0.001, OR: 1.21, 95%-CI: 1.157–1.266; Figure 4), the volume of demarcation (*p* = 0.003, OR: 0.997, 95%-CI: 0.995–0.997; Figure 5) and the FDCT-ASPECTS (*p* < 0.001, OR: 0.922, 95%-CI: 0.883–0.962, Figure 6) as independent factors for the development of a hyperdense lesion in FDCT after successful EVT. Further details are shown in Table 2.

**Figure 7:**
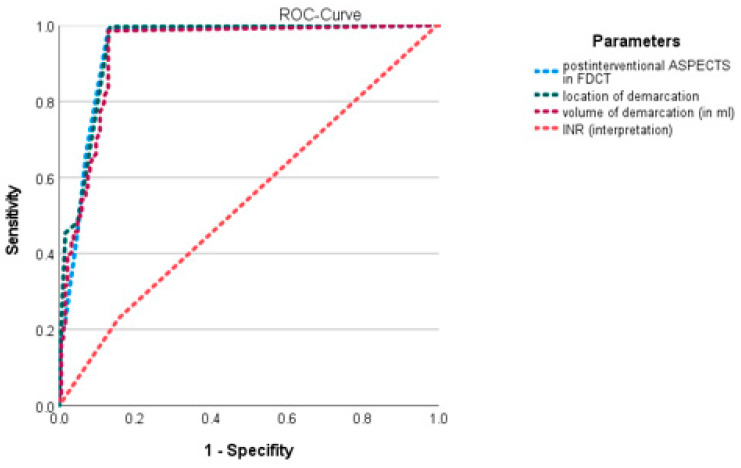
ROC curve of independent predisposing factors.

To further determine the predictive power of these items, a ROC curve analysis was performed (Figure 7). The AUC for the INR was 0.461 (*p* = 0.153, 95%-CI: 0.409–0.514), for the location of demarcation it was 0.053 (*p* < 0.001, 95%-CI: 0.03–0.076), for the volume of demarcation it was 0.067 (*p* < 0.001, 95%-CI: 0.04–0.093) and for the FDCT-ASPCETS it was 0.941 (*p* < 0.001, 94%- CI: 0.409–0.514).

## 4. Discussion

To our knowledge, we are the first to assess the predisposing factors for the development of hyperdensities in FDCT imaging after EVT.

For this analysis, we correlated research data regarding demographics, data regarding past medical history, stroke assessment and stroke treatment as well as short- and long-term follow-up data with the appearance of hyperdense areas in FDCT after successful EVT of LVO. Significant differences were only present in our patient cohort for NIHSS at admission, the time window, the haemostatic parameters (INR and aPTT), ASPECTS in initial NECT, the location of the most proximal LVO, CT perfusion parameters (infarct core, penumbra, mismatch ratio), the duration of EVT, the number of EVT attempts, the TICI score (TICI 2B vs. TICI 3), the affected brain region (grey matter vs. white matter), the volume of demarcation, FDCT-ASPECTS and the mRS at 90 days. Out of these test items, only the extension of these lesions (in total volume as well as in the applied ASPECTS), the location of the ischemic area (white vs. grey matter) and the plasmatic coagulation system could be demonstrated as independent factors for the development of such hyperdense lesions.

Furthermore, our results showed a prognostic association between the presence of these hyperdense lesions and the final extension of the infarct as well as the secondary rate of ICH development, as already demonstrated in previous publications [16,17,18,19,20,21,22,23].

Pathophysiologically, the phenomenon of the presence of hyperdense areas after EVT is discussed as a disruption of the blood–brain barrier (BBB) with subsequent leakage and extravasation of contrast media administered during the previous EVT [24,25,26,27,28,29].

Based on this pathophysiology, the extension of the lesion—represented by the total volume as well as the ASPECTS—is an obvious predisposing factor for the development of a leakage in the BBB and the development of hyperdensities as a larger damaged volume has a higher probability for a breakdown of the BBB. Furthermore, our data imply that the development of hyperdense lesions is more likely in the grey matter (basal ganglia and cortex) than in white matter. This represents the fact that grey matter has a higher level of vascular dependency regarding anatomical vascular density as well as physiological demand and is therefore more vulnerable to ischemic episodes and early damage of the BBB [30,31,32,33].

The plasmatic coagulation was identified as a third independent factor for the development of a hyperdensity after EVT in LVO. Regarding the plasmatic coagulation system, the results for INR were statistically significant (*p* = 0.003), whereas the results for aPTT showed a statistical trend (*p* = 0.063). Both imply that an interaction of BBB damage with the plasmatic coagulation system leads to a higher likelihood for contrast extravasation. To our knowledge, there has been no specific research regarding the topic of interaction between the plasmatic coagulation system and BBB integrity in the context of the development of hyperdense lesions after EVT, but research regarding the extension of ICH in the dependency of anticoagulation (which manipulates the plasmatic coagulation system) is a possible explanation base. In focusing on INR as the significant item in our results regarding the plasmatic coagulation, the work of Won et al. is especially interesting as it shows more contrast extravasation in dual-energy CT in combination with warfarin therapy, which is directly monitored via INR [34,35,36,37]. Therefore, the combination of a damaged BBB and reduced functionality of the plasmatic coagulation system can be explained in analogy to haemorrhagic lesions as a predisposing factor for contrast extravasation in ischemic areas.

Also very interesting are the parameters, which—in our analysis—do not show statistical difference or influence on the development of hyperdense lesions. Especially interesting is that neither an unknown time window, nor the time from symptom onset to hospitalisation, nor the duration of the EVT itself were identified as independent prognostic items for hyperdensities although our sub-populations differed in the initial time window as well as the duration of the EVT itself. Therefore, it seems that for the breakdown of the BBB in our study population, the affected volume dominates the time factor for the visual appearance of hyperdense areas after mechanical thrombectomy.

A second curiosity is the lack of a statistical relationship between the volume of contrast and the visualisation of hyperdensities. In our institute, contrast is always diluted with physiologic saline solution before, i.a., administration and documented as the maximal possible used volume. Therefore, we performed the statistical analysis with the maximal possible amount of administered contrast. Nonetheless, we could not demonstrate a statistical difference in the amount of applied contrast between our subgroups nor identify the volume of contrast as an independent factor for the development of hyperdensities although the neurotoxical potential of radiographic contrast and its influence on the integrity of the BBB is well known [38,39]. In addition, neither the methods of mechanical thrombectomy, the necessity of additional stent-angioplasty nor the IV application of rtPA as pharmaceutical therapy for AISs differed between the two groups and could therefore be identified as an independent influencing factor for the development of hyperdensities. In this context especially, thrombolytic medication such as rtPA is known to weaken the BBB. As this pathophysiological pathway is heavily supported by the presence of hypertonic blood pressure conditions—a factor also not differing in our data—the potential underestimation of the influence of the components of the treatment itself, as well as the influence of RR values, are probably based on the equal distribution across our study population [39].

### Limitations

Our study has several limitations:

First, our data are based on a single-centered retrospective study design and included only patients with successful EVT (meaning TICI ≥ 2B), which bears the risk of a selection bias. We focused on patients with successful EVT for our research as residual occlusion of large parts of the analysed vascular territory makes factors such as accessibility and collateral status of the ischemic areal highly unpredictable.

Secondly, we carried out our analysis on FCDT as the acquisition is standard procedure in our institute and—as it is conducted immediately after EVT—guarantees independency of the time factor (e.g., due to secondary transports between the angiographic suite and the CT). In doing this, we were fully aware that FDCT does not strictly follow the technical line of a traditional NECT regarding the HU scale and that, due to this, no commercially available automated approach of parenchymal evaluation on non-enhanced FDCT-data currently exists [40,41,42,43].

Thirdly, our control-group largely consisted of patients without visual infarct demarcation directly after EVT and not only of patients with classical hypodense lesions. This factor reflects real-world data as in many patients the treatment used restricts the infarct to a minimal or even non-detectable size although this may lead to an overestimation of the influence of volume-associated test items in our data.

## 5. Conclusions

Our results strongly support the association between hyperdense lesions after EVT in patients who have suffered AISs and LVO with the extension of the final infarct volume and the risk for the development of a secondary ICH as discussed in previous publications. For the development of these hyperdense lesions after EVT we could identify the extension of these lesions, the location in grey matter and the plasmatic coagulation systems as individual predisposing factors. These results may support future patient management by individualising therapeutic decisions in an early post-EVT phase.

## Figures and Tables

**Table 1:** Demographic and clinical characteristics.

Caracteristica	Total Study Population (n = 474 Patients)	No Hyperdensity in FDCT (n = 186 Patients)	Hyperdensity in FDCT (n = 288 Patients)	*p* Value
Demographics				
Age (in years)	75.2 [61.9–82.2]	74.9 [61.8–81.5]	72.4 [62–82.7]	0.549
Gender				
*Female*	266 (56.1%)	103 (55.4%)	163 (56.6%)	0.794
*Male*	208 (43.9%)	83 (44.6%)	125 (43.4%)	
Past medical history				
arterial hypertension	375 (79.2%)	145 (78%)	229 (79.5%)	0.712
Diabetes	143 (30.1%)	56 (30.1%)	86 (29.9%)	0.963
Medication				
*Anti-platelet therapy*				
Mono-therapy	126 (26.8%)	54 (29%)	72 (25%)	0.409
Dual-therapy	11 (2.3%)	4 (2.2%)	7 (2.4%)	
*Anticoagulation*	93 (19.5%)	30 (16.1%)	62 (21.5%)	0.145
Stroke assessment				
Initial mRS	0 [0–2]	0 [0–2]	0 [0–2]	0.248
NIHSS at admission	15 [11–19]	14 [10–19]	16 [12–20]	<0.001
Wake-up stroke	38 (8%)	16 (8.6%)	22 (7.6%)	0.706
Known time window (in min)	132 [65.3–365.3]	96.5 [60–263.3]	173 [68.8–426.3]	0.002
Chemical analysis				
*INR*	1.1 [1.0–1.1]	1.1 [1.0–1.2]	1.1 [1.0–1.3]	0.019
*INR (rating)*				
Low (<0.85)	1 (0.2%)	1 (0.5%)	0 (0%)	0.046
Normal (0.85–1.15)	377 (79.9%)	156 (83.9%)	223 (77.4%)	
High (>1.15)	94 (19.9%)	29 (15.6%)	65 (22.6%)	
*aPTT (in s)*	29.4 [26.9–32.6]	29.2 [26.7–32.0]	29.7 [26.9–33]	0.168
*aPTT (rating)*				
Low (<20 s)	0 (0%)	0 (0%)	0 (0%)	0.016
Normal (20–40 s)	446 (94.1%)	181 (97.3%)	265 (92%)	
High (>40 s)	28 (5.9%)	5 (2.7%)	23 (8%)	
*Platelet count (in 10³ platelets/µL)*	222 [181.3–271.5]	226.5 [184.8–268]	216 [180.8–272]	0.684
*Platelet count (rating)*				
Low (<150 × 10³ platelets/µL)	45 (9.5%)	15 (8.1%)	30 (10.5%)	0.716
Normal (150–400 × 10³ platelets/µL)	415 (87.5%)	167 (89.8%)	248 (86.1%)	
High (>400 × 10³ platelets/µL)	14 (3%)	4 (2.2%)	10 (3.5%)	
Stroke imaging				
*ASPECTS in NECT*	9 [7–10]	9 [8–10]	8 [7–10]	<0.001
*Side of occlusion*				
Left	232 (48.9%)	92 (49.5%)	140 (48.6%)	0.856
Right	242 (51.1%)	94 (50.5%)	148 (51.4%)	
*Location of occlusion (most proximal)*				
ICA	140 (29.5%)	35 (18.8%)	105 (36.5%)	0.001
M1	249 (52.5%)	115 (61.8%)	134 (46.5%)	
M2	85 (17.9%)	36 (19.4%)	49 (17%)	
*CT-perfusion*	331 (68.8%)	142 (76.3%)	189 (65.6%)	0.013
Infarct core in CT-P (in mL)	5 [0–29.3]	0 [0–12]	12 [0–41]	<0.001
Penumbra in CT-P (in mL)	123 [80–172.5]	108 [56–166]	133 [92–186]	0.007
Volume of mismatch in CT-P (in mL)	100 [64.5–145]	97.5 [54–146]	103 [68–144]	0.489
Mismatch-ratio in CT-P	4.8 [2.9–10.5]	8.2 [4.3–12.5]	4 [2.8–9.5]	0.005
Stroke treatment				
i.v. thrombolysis	316 (66.8%)	133 (71.5%)	183 (63.5%)	0.081
EVT related				
*Duration of EVT (in min)*	66 [44.8–99.3]	55.5 [9–82.5]	72 [48.3–107.8]	<0.001
*PTA/Stentangioplastia*	58 (12.2%)	18 (9.7%)	40 (13.9%)	0.172
*Typ of EVT intracranial*				
Aspiration	140 (29.5%)	57 (30.6%)	83 (28.8%)	0.266
Stentretrieving	101 (21.3%)	45 (24.2%)	56 (19.4%)	
Aspiration and stentretrieving	233 (49.2%)	84 (45.2%)	149 (51.7%)	
*Number of EVT attempts*	2 [1–3]	2 [1–3]	2 [1–3]	0.004
*KM volume (in ml)*	150 [100–200]	150 [100–200]	150 [100–200]	0.062
*KM volume (grouped)*				
<100 mL	150 (31.6%)	63 (33.9%)	87 (30.2%)	0.059
100–150 mL	139 (29.3%)	61 (32.8%)	78 (27.1%)	
150–200 mL	151 (31.9%)	54 (29%)	97 (33.7%)	
>200 mL	34 (7.2%)	8 (4.3%)	26 (9%)	
*TICI*				
2B	111 (23.4%)	33 (17.7%)	78 (27.1%)	0.019
3	363 (76.6%)	153 (82.3%)	210 (72.9%)	
*RR during EVT*				
Mean arterial pressure (in mmHg)	94.9 [87–100.8]	94.8 [86.8–100.8]	95.5 [87.3–101]	0.74
Min. systolic RR (in mmHg)	115 [100–130]	120 [105–125]	115 [100–130]	0.463
Max. systolic RR (in mmHg)	150 [140–170]	150 [140–165]	150 [140–170]	0.358
Min. diastolic RR (in mmHg)	65 [60–70]	65 [60–75]	60 [60–70]	0.081
Max. diastolic RR (in mmHg)	85 [80–95]	85 [80–95]	85 [80–95]	0.152
FDCT after EVT				
Location of demarcation				
*Basal ganglia*	165 (34.8%)	16 (8.6%)	149 (51.7%)	<0.001
*White matter*	14 (3%)	6 (3.2%)	8 (2.8%)	
*Cortical*	70 (14.8%)	2 (1.1%)	68 (23.6%)	
*Mixed pattern*	64 (13.5%)	1 (0.5%)	63 (21.9%)	
Volume of demarcation (in mL)	2.8 [0–13.8]	0 [0–0]	25.1 [2.8–33.7]	<0.001
FDCT-ASPECTS	9 [7–10]	10 [10–10]	8 [7–9]	<0.001
Follow-up				
Follow-up NECT (24–48 h after EVT)				
*ICH*	49 (10.3%)	9 (4.8%)	40 (13.9%)	0.002
*Volume of demarcation*	26 [6–96]	8 [0–27.9]	54.5 [18–130]	<0.001
mRS at 90 days	4 [2–5]	3 [2–4]	4 [3–6]	<0.001

**Table 2:** Results of multivariable regression analysis.

Caracteristics	*p* Value	Odds Ratio	95% Confidence Interval
Stroke assessment			
chemical analysis			
*INR (rating)*	0.003	1.212	1.067–1.379
*aPTT (rating)*	0.063	1.372	0.982–1.914
known time window (in min)	0.715	1	1–1
Stroke imaging			
*ASPECTS in NECT*	0.619	0.993	0.967–1.020
*Location of occlusion (most proximal)*	0.26	0.969	0.915–1.024
*CT-perfusion*			
Infarct core in CT-P (in ml)	0.391	0.999	0.998–1.001
Penumbra in CT-P (in ml)	0.515	1	1–1
Mismatch-ratio in CT-P	0.918	1	0.994–1.005
Stroke treatment			
Duration of EVT (in min)	0.952	1	0.999–1.002
Number of EVT attempts	0.415	0.987	0.958–1.018
TICI	0.608	0.971	0.869–1.085
FDCT after EVT			
*Location of demarcation*	<0.001	1.21	1.157–1.266
*Volume of demarcation (in ml)*	0.003	0.997	0.995–0.999
*FDCT-ASPECTS*	<0.001	0.922	0.883–0.962
Follow-up NECT (24–48 h after EVT)			
Volume of demarcation	0.399	1	0.999–1
ICH	0.445	0.715	0.612–0835

## Data Availability

The datasets generated and analysed during the current study are available from the corresponding author on reasonable request.
